# Edaravone alleviated propofol‐induced neural injury in developing rats by BDNF/TrkB pathway

**DOI:** 10.1111/jcmm.16422

**Published:** 2021-05-01

**Authors:** Yangliang Yang, Jing Yi, Mengzhi Pan, Baoji Hu, Hongwei Duan

**Affiliations:** ^1^ Department of Anesthesiology Shanghai Pudong Hospital Fudan University Shanghai China

**Keywords:** edaravone, hippocampus, propofol, the brain‐derived neurotrophic factor, TrkB

## Abstract

As a variety of free radical scavenger, edaravone has shown its potential in producing antioxidant, anti‐inflammatory and neuroprotective effects in various disease models. However, the underlying mechanism behind the neuroprotective effects of edaravone remained unclear. This study is aimed at determining the effects of edaravone on neuroprotection and anti‐inflammatory through a propofol‐induced neural injury rat model. Firstly, an observation was made of apoptosis and neuroinflammation in the hippocampus of developing under the influence of propofol. It was found out that propofol could produce inflammatory effects in the hippocampus by enhancing the astrogliosis (GFAP) activation and elevating the level of neuronal nitric oxide synthase (nNOS), pro‐inflammatory cytokines interleukin‐6 (IL‐6) and tumour necrosis factor‐α (TNF‐α). Meanwhile, the increase of apoptosis cells and the decrease of neurons (NeuN) were speculated to aggravate neural injury. Furthermore, it was demonstrated that edaravone intervention can reverse the neural apoptosis and inflammation. Additionally, the intraperitoneal injection of edaravone, the intraperitoneal injection of the brain‐derived neurotrophic factor (BDNF)‐mimicking small compound (7,8 dihydroxyflavone) and the intracranial injection of the exogenous BDNF were all respectively effective in alleviating the propofol‐induced neural apoptosis and inflammation in the hippocampus. It was also found out that edaravone‐activated downstream signalling through tyrosine kinase receptor B (TrkB) receptors in astrocyte, microglia and neuron. However, the neural injury of propofol had no impact on long‐term learning and memory, except causing a short‐term neurotoxicity. In conclusion, edaravone could alleviate the propofol‐induced neural injury in developing rats through BDNF/TrkB pathway.

## 1 INTRODUCTION

General anaesthesia is regarded as the most significant part of surgery, especially in newborns.^1^, ^2^ In recent studies, it has been reported that there is a possibility that general anaesthetics could hinder brain development and reduce the scores in neurobehavioral development.^2^, ^3^, ^4^ Therefore, it is considered necessary to establish whether the over‐dose use of anaesthetics can lead to neural injury in rodents.

Propofol (2,6‐diisopropylphenol), an intravenous anaesthetic applied as a gamma‐aminobutyric acid A (GABAA) receptor agonist and an N‐methyl‐D‐aspartate receptor (NMDAR) antagonist, has demonstrated an excellent sedative‐hypnotic effect that is beneficial to surgery and ICU treatment.^5^ However, there are some recent studies suggesting that propofol can lead to cell death in cortical cells, hippocampal neurons and neural stem cells.^6^, ^7^ In addition, the evidence has emerged that propofol can also increase the level of reactive oxygen species (ROS) and interleukin‐1β (IL‐1β), thus inducing neuroinflammation.^7^ Numerous studies have demonstrated that cellular apoptosis, the generation of excess ROS, inflammatory mediators and mitochondrial injury are the primary mechanisms behind the propofol‐induced neuronal cell death found in hippocampus.^8^ However, the exact mechanisms of propofol‐induced neural injury and apoptosis are still unclear.

As an effective free radical scavenger, edaravone (3‐methyl‐1‐phenyl‐2‐pyrazolin‐5‐one) can lead to antioxidant effects by reducing lipid peroxidation.^9^, ^10^ Edaravone can also produce neuroprotective effects in such animal models as diabetic stroke, ischaemic reperfusion injury, lipopolysaccharide‐induced abnormal behaviour, Alzheimer's disease, Parkinson's disease and amyotrophic lateral sclerosis.^11^ In addition, cerebral ischaemia/ reperfusion (I/R) injury could induce mitochondrial dysfunction, which can lead to an increase in the production of ROS.^12^ The oxidative stress caused by cerebral I/R can activate inflammatory pathway through the activation of glial cells (microglia, astrocyte).^13^ Besides, there are some recent studies demonstrating the potential of edaravone in alleviating neural cells apoptosis and cognitive dysfunction.^14^ Considering the roles played by edaravone and the pathogenesis of propofol‐induced neural injury, it is natural to hypothesize that the application of edaravone can mitigate the adverse effects caused by propofol on the nervous system of developing rats.

Neurotrophins are the potential regulators of neuronal structure. For example, BDNF, a protein mainly synthesized in the neuron, plays an important role in the survival, differentiation, growth and development of neuronal cells.^15^, ^16^ Furthermore, BDNF can increase the size and density of dendritic spine by activating its high‐affinity receptor, TrkB, which is also associated with dendritic spine homeostasis, promoting drug‐induced dendritic spin genesis, synapse formation, neuronal maturation and synaptic plasticity through the regulation of phospholipase C gamma (PLCγ)/phosphatidylinositol 3‐kinase (PI3K)/mitogen‐activated protein kinase.

(MAPK) signalling cascade.^17^, ^18^, ^19^ Meanwhile, the reduced level of TrkB was often reported in neural injury and the down‐regulation of long‐term potentiation of GABAergic synapses (LTPGABA) expression.^20^, ^21^ As a BDNF‐mimicking small compound, 7,8 dihydroxyflavone (7,8 DHF) is a member of the flavonoid family, and it is widely present in various vegetables and fruits. Besides, it can produce antioxidant and anti‐inflammatory effect, which is conducive to treating various neurological disorders, improving action‐outcome memory and triggering the same pro‐survival/growth signalling cascades as BDNF.^17^, ^18^, ^19^, ^22^ It is difficult for BDNF to penetrate the blood‐brain barrier (BBB). By contrast, 7,8 DHF can permeate the BBB after Systemic administration to perform the biological role of BDNF‐TrkB.^21^, ^23^, ^24^, ^25^ In addition, DHF is considered a promising therapeutic agent for treating various neurodegenerative diseases, such as Parkinson's disease and Alzheimer's disease.^22^, ^23^, ^24^ In our research, the accuracy of the pathway was also proved through exogenous BDNF. In this study, it is reported that intraperitoneal injection propofol in P7 rats can cause neural injury to hippocampus and that BDNF/TrkB pathway is associated with propofol‐induced neural apoptosis and inflammation.^7^, ^25^ In addition, edaravone can alleviate propofol‐induced neural injury via BDNF/TrkB pathway.

## 2 MATERIALS AND METHODS

### 2.1 Animals

**FIGURE 1:**
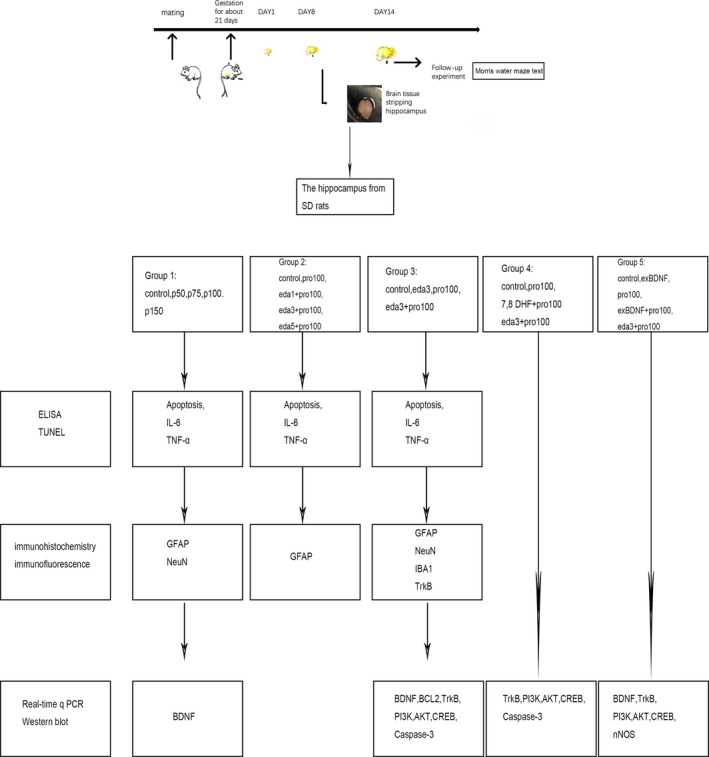
The experimental procedure. IL‐6, Cytokines interleukin‐6; TNF‐α, Tumour necrosis factor‐α; GFAP, Glial fibrillary acidic protein; NeuN, Neuronal marker; IBA1, Ionized calcium‐binding adaptor molecule 1; BDNF, Brain‐derived neurotrophic factor; BCL2, B cell lymphoma‐2; TrkB, Tyrosine kinase receptor B; PI3K, Phosphatidylinositol 3‐kinase; AKT, Protein kinase B; CREB, cAMP‐response element‐binding protein; nNOS, Neuronal nitric oxide synthase

All experimental procedures were approved by the Pudong hospital Animal Care and Use Committee at Fudan university. 139 P7 Sprague Dawley neonatal pups (weight = 15‐18g, Fudan university, China, Shanghai) were randomly divided into Control group (C) (injected with intralipid, n = 13), propofol at different concentrations (injected with propofol 50,75,100,150 mg/kg, n = 13 for each), edaravone intervention at different concentration (injected with 100mg/kg propofol and edaravone in 1,3,5 mg/kg and only edaravone in 3mg/kg, n = 13 for each), 7,8 DHF + pro100 (injected with 100mg/kg propofol and 5mg/kg of 7,8 DHF group, n = 3) and exBDNF + pro100 (injected with 100mg/kg propofol and 1μl exogenous BDNF group, n = 4). The control group was injected with the 150mg/kg of normal intralipid, and rats in the pro100 or pro150 group were initially intraperitoneally injected with half dose of propofol, and when their righting reflex recovered (30‐50 min), another was injected.^2^ The edaravone intervention group was intraperitoneally injected 30 min before propofol injection. 7, 8 DHF dissolved in 1% DMSO solution was intraperitoneal injection 30 min before propofol injection.^21^ For the administration of exogenous BDNF, stereotaxic apparatus was used for injection into hippocampus. 7‐day rats were anesthetized with 4% chloral hydrate. After complete anaesthesia, the animal was fixed in the stereotaxic apparatus that the head is tight. Cut the skin along the midline of the skull. We tested that aligned the needle of the instrument with the marked injection point precisely and then adjusted the coordinates of −2 mm posterior bregma, ±2 mm bilateral and 1.5 mm deep skull. We marked the coordinates and drilled a small hole in it. Each rat was injected with Human/Murine/Rat BDNF (450‐02‐10, PeproTech, China, 50ng/ml) 1μl (Figure 1).

### 2.2 Western blot

After 6 hours of interval anaesthesia, the tissues of the hippocampal were removed, and the tissues in each group were added to ice‐cold the radio immunoprecipitation assay (RIPA, Boster, Wuhan, China) buffer containing the protease inhibitor phenylmethanesulfonyl fluoride in added protease inhibitor. The completed homogenates were centrifuged at 12 000 r/min at 4°C for 20 minutes, and then, the protein concentration in the supernatant was determined using the bicinchoninic acid (BCA, Boster, Wuhan, China) method. After adjusting the same concentration of each group, electrophoresis was performed to obtain the separated protein bands. The protein of each experimental group was mixed with an equal volume of loading buffer and boiled for 3 min. Equal amounts of protein from each sample were separated via 12% sodium dodecyl sulphate‐polyacrylamide gel electrophoresis (SDS–PAGE) and were transferred to polyvinylidene fluoride (PVDF) membranes. Membranes containing the transferred proteins were blocked with 5% skim milk in TBST (TBS, pH 7.5, containing 0.1% Tween‐20) for 2h at room temperature (RT) and then incubated with a dilution of primary antibody (anti‐BDNF, anti‐phosphorylated TrkB, anti‐TrkB, anti‐phosphorylated‐protein kinase B (AKT), anti‐AKT, anti‐phosphorylated‐cAMP‐response element‐binding protein (CREB), anti‐CREB, anti‐caspase 9, anti‐caspase 3, anti‐GFAP, anti‐NeuN, anti‐IBA1 purchased by Abcam, anti‐neuronal nitric oxide synthase (nNOS) purchased by ABclonal) at 4°C. The membranes were washed thoroughly with TBST for three times and incubated with horseradish‐peroxidase‐conjugated secondary antibody (Proteintech, China; dilution 1:5000) for 60min at RT. Finally, the optical densities of the resultant bands were recorded and analysed using C‐Digit software (LI‐COR, USA). The relative expression was normalized to GAPDH (Proteintech, China).

### 2.3 Enzyme‐linked immunosorbent assay (ELISA) of pro‐inflammatory factors (IL‐6, TNF‐α)

Pro‐inflammatory factors (IL‐6, TNF‐α) in the hippocampus was measured using rat ELISA kit (ERC003.96, NeoBioscience, Shenzhen, China; ERC102a.96, NeoBioscience, Shenzhen, China). The one of hippocampus tissues from same sample was ground and homogenized, then followed by centrifugation (5000 rpm) for 10 min at 4°C to suck out the supernatant. The IL‐6 and TNF‐α ELISA kits were detected at 450nm from instructions.^6^

### 2.4 Real‐time quantitative PCR

Total RNA was extracted with the TRIzol kit (Invitrogen, China). cDNA synthesis was performed according to the manufacturer's instructions. Real‐time PCR was performed using the Veriti DX (Life). The following primers were used: BDNF‐ forward primer (5’‐TGAGCCGAGCTCATCTTTGC‐3’), reverse primer (5’‐ATAGCGGGCGTTTCCTGAAG‐3’); B cell lymphoma‐2 (BCL2) forward primer (5’‐AAGCTCAGAGGGGCTA‐3’), reverse primer (5’‐CAGGCTGGAAGGAGAAGATG‐3’); and GAPDH forward primer (5’‐TGCCACTCAGAAGACTGTGG‐3’), reverse primer (5’‐TTCAGCTGGGATGACCTT‐3’). The products of the PCR were analysed by the Roche480II Real‐Time PCR System (Roche), and GAPDH was used as an endogenous control. The results were calculated by 2^^‐⊿⊿Ct^, where Ct is the threshold cycle for each transcript.

### 2.5 Immunohistochemistry analysis

7‐day‐old rats were deeply anesthetized with 10% chloral hydrate and fixed by cardiac perfusion with 4% paraformaldehyde for 30 minutes (n = 6 per group). The brain was removed, fixed in 4% paraformaldehyde overnight, dehydrated in ethanol and xylene, and then embedded in paraffin. In the hippocampal area appearing, 30 brain slides were consecutively cut from each animal which were used for TUNEL, immunohistochemistry and immunofluorescence assay. Brain was cut coronally into 4μm thick and mounted on a glass slide. To improve antigen recovery, dewaxed and rehydrated sections were maintained at high temperature and pressure (10 minutes in 0.1 M citrate buffer, pH 6.0) to maintain antigen recovery and incubated with 3% H2O2 to block endogenous peroxidase. After washing in distilled water (dH_2_O) and phosphate buffer saline (PBS), the sections were immunolabelled with a primary antibody against IBA1 (rat monoclonal, 1:400, Cell Signaling Technology, USA) at 4°C overnight. After washing in PBS, the sections were exposed to secondary antibodies for 10 minutes at RT and then rinsed in PBS. Sections were then visualized with diaminobenzidine (DAB), counterstained with haematoxylin and imaged on an Olympus CX41 microscope. Five fields were randomly measured from each slide, then averaged and represented by optical density. The integrated optical density (IOD) of DAB‐positive cells were analysed by ImageJ (National Institutes of Health, Bethesda, MD).^26^

### 2.6 Immunofluorescence staining

7‐day‐old rats were anesthetized and sacrificed, and then, brain tissue was removed for immunofluorescence staining. Sections were treated with 0.4% Triton‐100 (Solarbio, China) for 30 minutes and blocked with normal donkey serum (Servicebio, China) for 1.5 hours at room temperature. Sections were then left overnight at 4°C in rat anti‐NeuN, rat anti‐GFAP (rat monoclonal, 1:1000, Cell Signaling Technology, USA), rat anti‐IBA1, rat anti‐TrkB (rat monoclonal, 1:400, Cell Signaling Technology, USA). The next day, the sections were incubated in a mixture of fluorescein‐conjugated anti‐goat IgG and anti‐rat IgG and then left in the dark at 37°C for 2 hours. Nuclei were stained with 4,6‐diamidino‐2‐phenylindole (DAPI) for 15 minutes at room temperature. All microphotographs were taken under a laser scanning confocal microscope.^27^ Pixels were assessed quantitatively by ImageJ. Statistical analysis was performed using Prism 8 (GraphPad Software, La Jolla, CA).

### 2.7 TUNEL staining

The sections of the apoptosis of hippocampal neural cells were detected by TUNEL method using the apoptosis detection kit (Roche In Situ Cell Death Detection Kit, USA). Five fields of view were randomly selected under 50‐fold, 200‐fold and 400‐fold optical microscope. The normal cell nucleus was blue, and the apoptosis‐positive cells were brown or yellow. The total number of cells and the number of apoptotic cells were calculated then the average value was recorded to reflect apoptosis. Apoptosis rate = apoptotic cell number/total cell number × 100%.

### 2.8 Arterial blood gas assessment

At the end of anaesthesia, a 32‐gauge hypodermic needle was used to obtain arterial blood samples from the left ventricle and analysed by a blood gas analyser. Immediately after blood collection, the arterial hydrogen ion concentration (pH), arterial carbon dioxide tension (PaCO_2_), arterial blood oxygen partial pressure (PaO_2_) and arterial blood oxygen saturation (SaO_2_) were measured. In all situation, the process was completed in less than 5 minutes.^26^

### 2.9 Morris water maze test

On day 21 after modelling, Morris water maze test was used to evaluate spatial learning and memory of rats (n = 5 for each). The experimental apparatus was a circular pool (150 cm in diameter and 50 cm in height, containing water at 28°C) with water coloured by blue ink and divided into 4 equally quadrants (S1, S2, S3 and S4). The pool was placed in a test room containing different prominent visual cues in each quadrant. Within 60 seconds, each rat was randomly placed in one of the four starting quadrants, facing the pool wall, looking for a hidden circular platform (diameter 11 cm, 1‐2 cm underwater). Regardless of whether the rat found the platform or swam in the water for 60 seconds, we placed it on the platform and rested for 20 seconds. Four tests were performed from each of the four quadrants every day for 5 consecutive days. The escape latency (EL) was defined as the time from the start of the recording to the finding of the platform. The average time calculated from the 4 quadrants in 5 days represents the performance of the rats during this process. On the sixth day, the platform was removed. The rats were allowed to swim in the water for 60 seconds, and the swimming time in the platform quadrant was recorded as the space exploration time (SET). The swimming lane, space exploration time and swimming speed for calculating escape latency are recorded through the video track.

### 2.10 Statistical analysis

The data are expressed and graphically analysed by means of the mean ± standard deviation (SD) of SPSS 25.0 software (SPSS IBM, USA) and Prism 8 software program (GraphPad software, USA). Arterial blood gas, immunofluorescence, Western blot and immunohistochemical analysis were analysed using one‐way analysis of variance (ANOVA) followed by appropriate Dunnett's post hoc tests. *P* values<0.05 were considered to be significant.

## 3 RESULTS

### 3.1 Physiological variables of developing rats

We investigated physiological parameters such as blood gas analysis during the experiment. As expected, there were no significant differences about PH, SaO_2_, PaO_2_ and PaCO_2_ in four experimental parts, indicating that the physiological signs of experimental animals and establishing animal models were stable. There was no interference from external factors (Table S1, *P*>0.05).

### 3.2 Propofol promotes neural apoptosis and neuroinflammatory in hippocampus of developing rats

**FIGURE 2:**
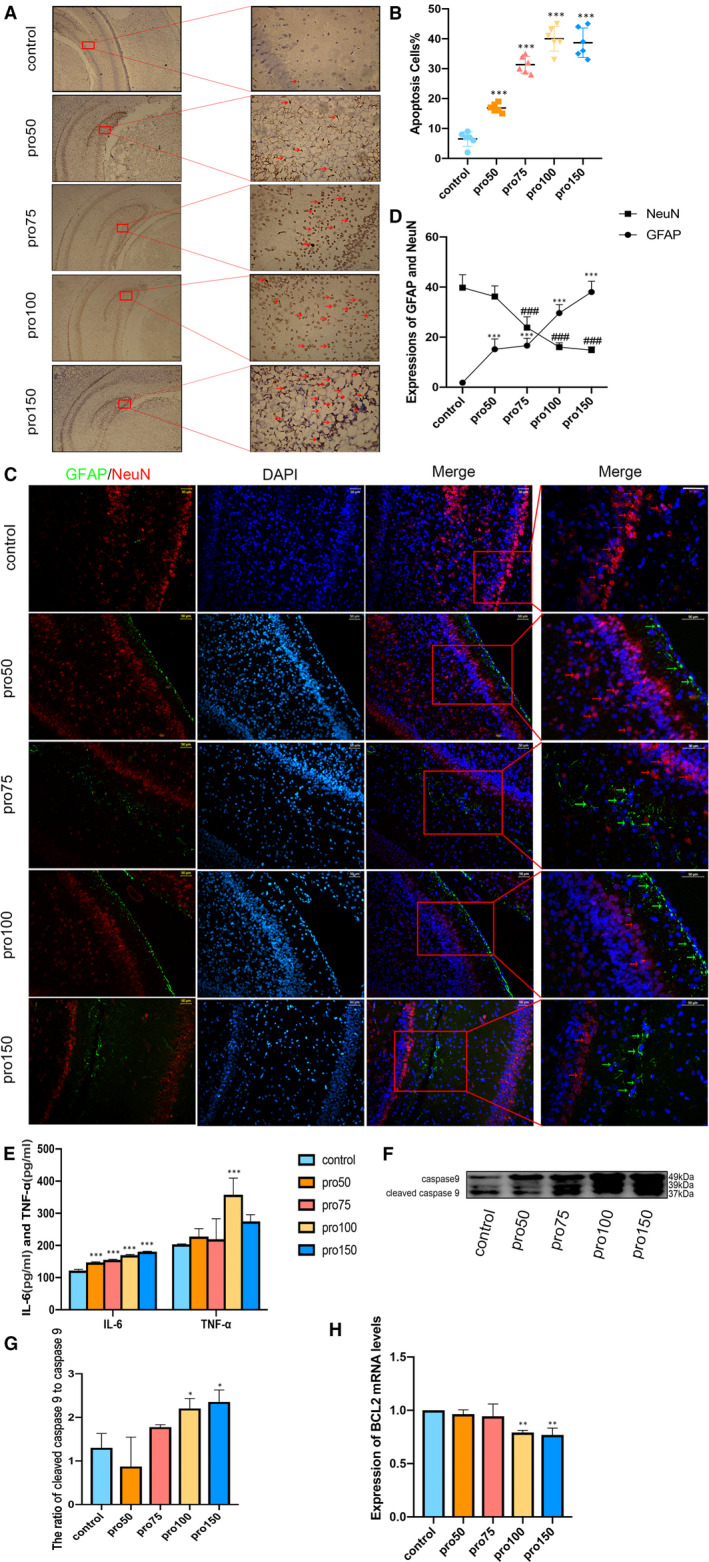
Propofol mediates neural apoptosis and neuroinflammatory in hippocampus of developing rats. A and B, hippocampal neuronal apoptosis in hippocampal DG region measured with TUNEL. C and D, GFAP (Green) and NeuN (Red) detected glial cells and neurons in propofol exposed to different concentrations. E, IL‐6 and TNF‐α were measured by ELISA. F and G, Western blot analysis detected protein levels of cleaved caspase 9. H, RT‐qPCR detected BCL2 expressions in hippocampus of developing rats. All data expressed as the mean ± SD. *P <.05, **P<.01, ***P<.001 as compared with control group. ^###^ *P*<.001 as compared with control group in expression of NeuN. IL‐6, Cytokines interleukin‐6; TNF‐α, Tumour necrosis factor‐α; BCL2, B cell lymphoma‐2; GFAP, Glial fibrillary acidic protein; NeuN, Neuronal marker. (n = 6 per group)

While with the addition of different concentrations of propofol (50, 75, 100, 150mg/kg), the number of TUNEL‐positive neurocytes was increased in the hippocampal DG region, which indicated increasing doses of propofol caused a up‐regulation in the number of apoptosis cells of hippocampus in P7 rats (Figure 2A, B, F4,25 = 110.7, *P*<0.001). Immunofluorescence staining was applied to detect the elevated astrocytes (GFAP) and decreased neurons of developing rat hippocampal cells (Figure 2C, D, F4,8 = 72.76, *P*<0.001), suggesting that changes in different proportions of astrocytes and neurons may affect neural development. Pro‐inflammatory factors IL‐6 and TNF‐α in developing rats after exposure to propofol were significantly increased when compared to control group (Figure 2E, F1,30 = 92.71, *P*<0.001). According to Western blot results, we found that after exposure to propofol, the expression of cleaved caspase 9 was significantly increased when compared to control group (Figure 2F, G, F4,10 = 8.25, *P*<0.05), suggesting that exposure to propofol could cause mitochondrial injury. Then, compared with group control, a decreased in BCL2 mRNA level was observed in group pro100 and group pro150 (Figure 2H, F4,20 = 0.4, *P*<0.01). From these findings, we hypothesized that propofol induced apoptosis, inflammatory mediators and mitochondrial injury in developing hippocampal rats.

### 3.3 Edaravone pre‐treatment reduces propofol‐induced neural apoptosis and neuroinflammation in hippocampus of developing rats, but it does not affect long‐term learning and memory ability

**FIGURE 3:**
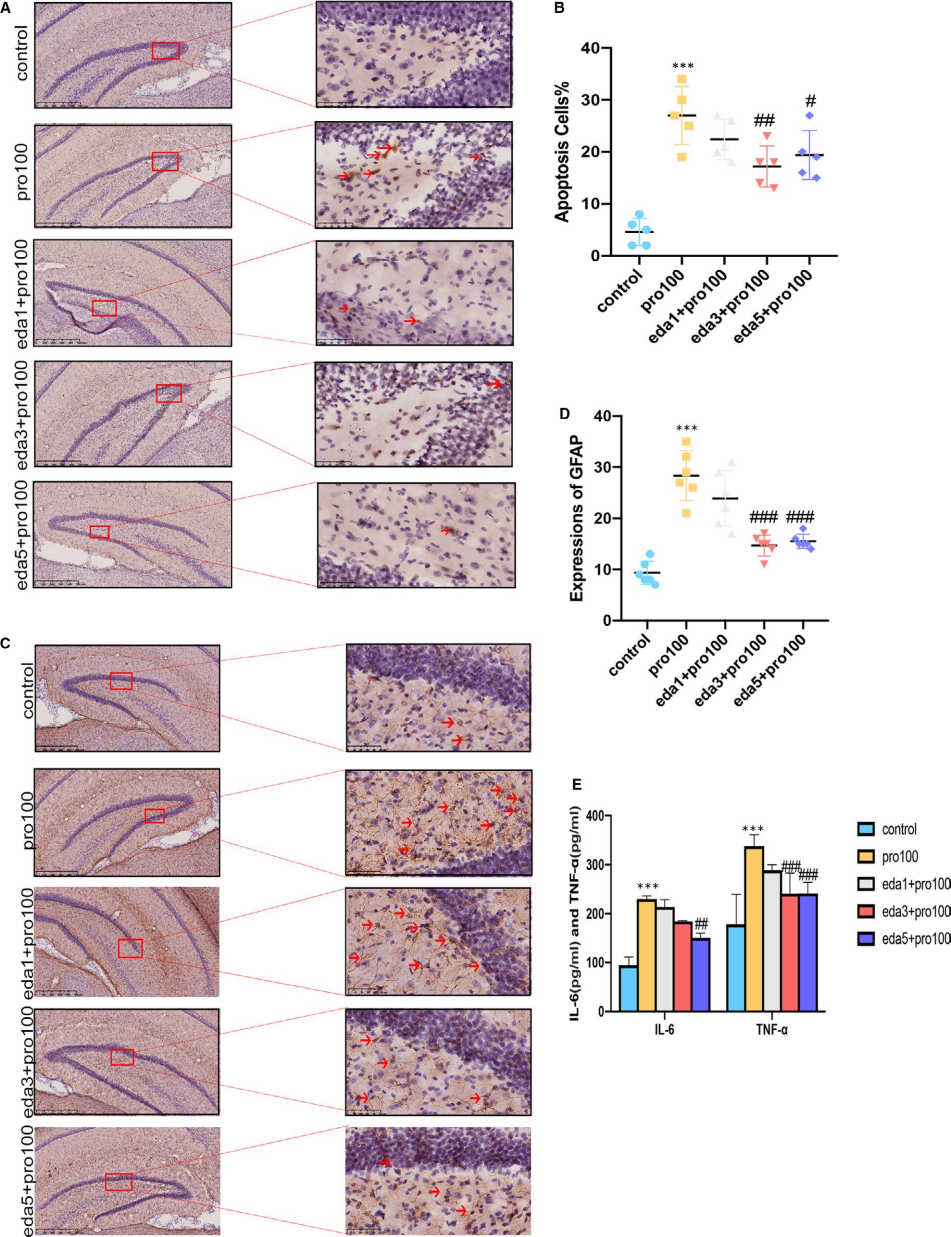
Edaravone pre‐treatment reduces propofol‐induced neural apoptosis and neuroinflammation in hippocampus of developing rats. A‐D, TUNEL and immunohistochemistry detected apoptosis cells and astrocytes in hippocampal DG region with edaravone intervention (n = 6 per group). E, IL‐6 and TNF‐α were measured by ELISA. All data expressed as the mean ± SD. ***P<.001 as compared with control group. ^#^ *P*<.05, ^##^ *P*<.01, ^###^ *P*<.001 as compared with pro100 group. IL‐6, Cytokines interleukin‐6; TNF‐α, Tumour necrosis factor‐α; GFAP, Glial fibrillary acidic protein

**FIGURE 4:**
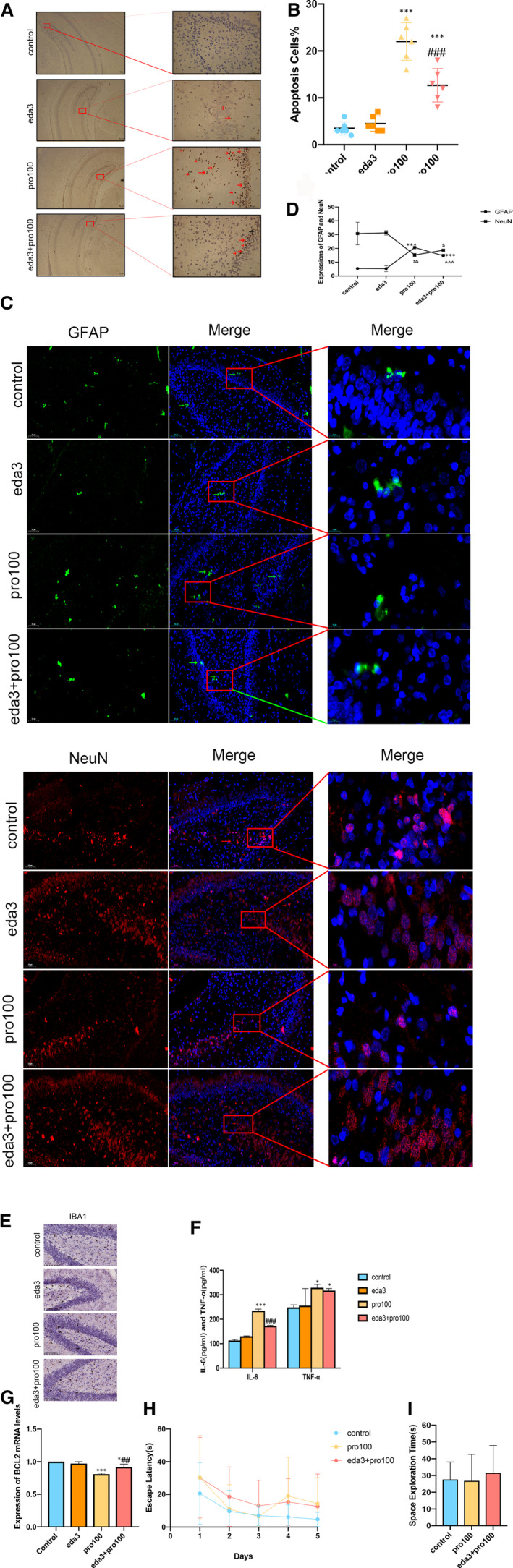
Edaravone pre‐treatment attenuates propofol‐induced hippocampal neural injury in developing rats and Morris water maze results. A‐D, TUNEL and immunofluorescence detected apoptosis cells astrocytes and neurons in hippocampal DG region with 3mg/kg edaravone intervention (n = 6 per group). E, immunohistochemistry detected microglia cells in hippocampal DG region with 3mg/kg edaravone intervention (n = 6 per group). F, IL‐6 and TNF‐α were measured with 3mg/kg edaravone intervention by ELISA. G, RT‐qPCR detected BCL2 expressions in hippocampus of developing rats. H and I, escape latency and space exploration time in three groups in MWM test (n = 5 per group). All data expressed as the mean ± SD. *P <.05, ***P<.001 as compared with control group. ^###^ *P*<.001 as compared with pro100 group. ^^^^^ *P*<.001 as compared with Pro100 group in GFAP;^$^ *P*<.05, ^$$^ *P*<.01 vs control group in NeuN. GFAP, Glial fibrillary acidic protein; NeuN, Neuronal marker; IBA1, Ionized calcium‐binding adaptor molecule 1; GFAP, Glial fibrillary acidic protein; NeuN, Neuronal marker; IBA1, Ionized calcium‐binding adaptor molecule 1; BCL2, B cell lymphoma‐2

In order to observe effect of edaravone, we established another model in P7 rats with propofol exposure and intervention of edaravone. Our results certified that 100mg/kg propofol significantly increased the number of apoptosis cells in hippocampus after 7‐day exposure (Figure 3A, B, F4,20 = 19.27, *P*<0.001). This concentration was further adopted to assess the effect of edaravone. Our study observed that rats with edaravone (1, 3, 5mg/kg) resulted into significant reduction in apoptosis cells when compared to group animals in propofol treatment. Furthermore, we then examined whether edaravone can exhibit a significant reduction in microglia and astrocyte to alleviate propofol‐mediated inflammation. In our study, in ELISA and Immunohistochemistry assays, we found that edaravone decreased glial cells (astrocytes) (Figure 3C, D, F4,25 = 26.58, *P*<0.001) and the pro‐inflammatory cytokines IL‐6 and TNF‐α (Figure 3E, F4,30 = 0.9853, *P*<0.01, *P*<0.001). The results indicated that edaravone alleviated propofol‐induced inflammatory in hippocampus by decreasing pro‐inflammatory cytokines and glial cells. Data shown in Figure 4A, B confirmed that when compared to pro100 group animals, hippocampal apoptosis cells from eda3 + pro100 group rats were significantly decreased (F_3,20_ = 53.35, *P*<0.001). As we observed massive activation of astrocyte and microglia in the hippocampus of propofol‐induced rats, we hypothesized whether edaravone is capable of alleviating the inflammatory reaction and increasing neurons. Then, we found that edaravone administration dramatically attenuated the number of astrocytes and microglia in the hippocampus of propofol‐induced rats (Figure 4C‐E, *P*<0.001). In addition, the number of neurons in eda3 + pro100 group rats was also increased in comparison with that in pro100 group (Figure 4D, *P*<0.05). Accordingly, we next investigated the protein levels of pro‐inflammatory (IL‐6 and TNF‐α) in hippocampal of rats. Edaravone treatment significantly attenuated the production of all test pro‐inflammatory cytokines in the hippocampus (Figure 4F, F3,24 = 1.443 *P*<0.001). By contrast, only exposure propofol significantly decreased the mRNA expression levels of BCL2 (Figure 4G, F3,16 = 12.27, P<0.001). Treatment with edaravone significantly reversed propofol‐induced change in the mRNA expression levels of BCL2 (*P<*0.01). To investigate the cognitive performance of three groups, we conducted the MWM test. The average of the latency to platform revealed no significant difference in each group (Figure 4H, *P*>0.05). On the sixth day, a probe test was observed to experiment the spatial memory retrieval. The results did not discover any differences in three groups (Figure 4I, *P*>0.05).

### 3.4 Edaravone pre‐treatment attenuates propofol‐induced hippocampal momentarily neural injury in developing rats by TrkB/PI3K/CREB pathway

**FIGURE 5:**
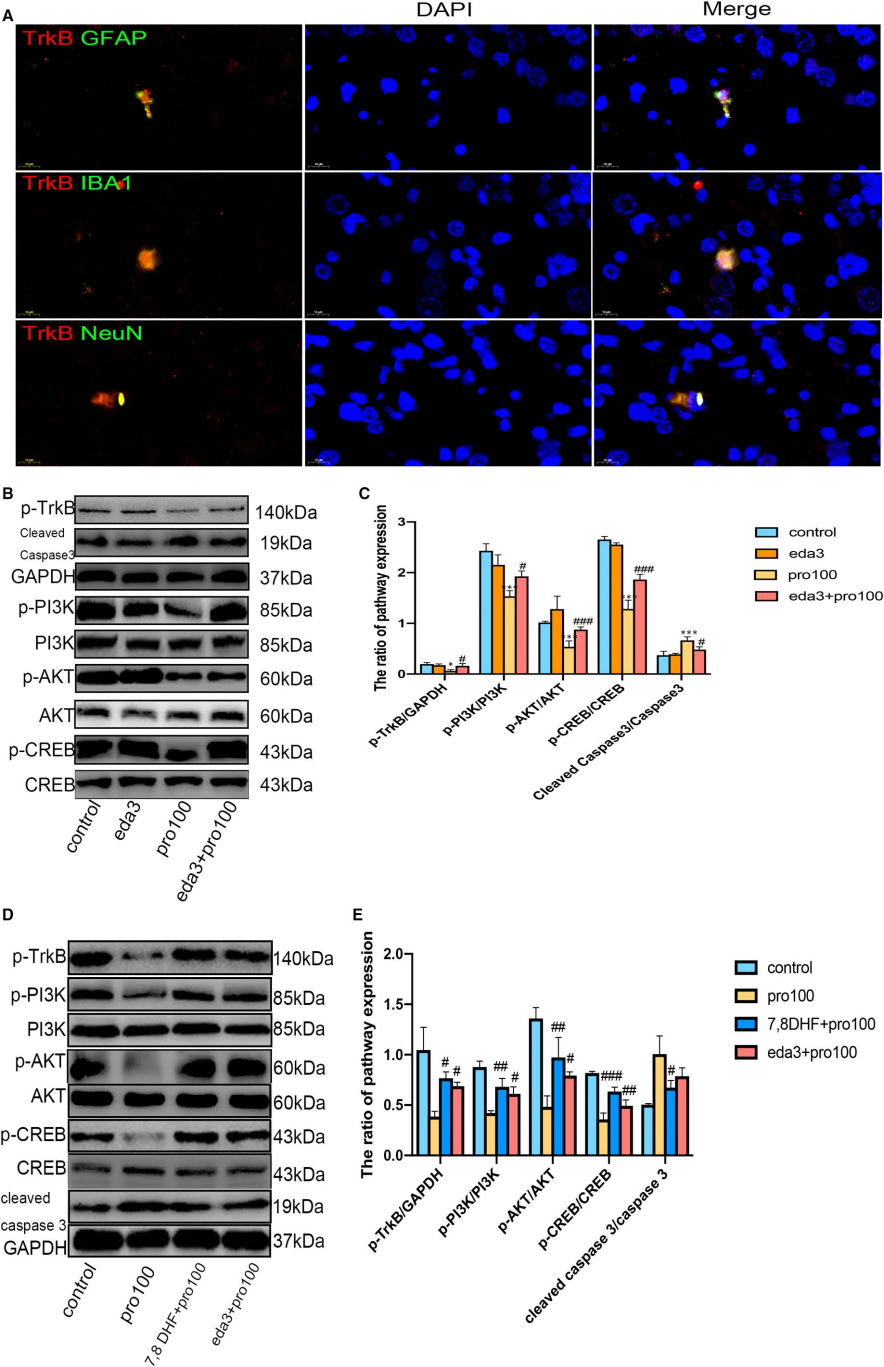
Edaravone and 7,8‐DHF pre‐treatment attenuates propofol‐induced hippocampal neuronal injury in developing rats by TrkB/PI3K/CREB pathway and immunofluorescence double staining results. A, Immunofluorescence co‐localization of TrkB (Red) with astrocytes (GFAP. Green), neurons (NeuN. Green) and microglia (IBA‐1. Green) in eda3 + pro100 group rats (n = 6 per group). B and C, Western blot analysis detected protein levels of p‐TrkB and the PI3K/AKT pathway‐related proteins with 3mg/kg edaravone intervention. D and E, Western blot analysis detected protein levels of p‐TrkB and the PI3K/AKT pathway‐related proteins with 7,8 DHF or 3mg/kg edaravone intervention. All data expressed as the mean ± SD. *P <.05, ***P<.001 as compared with control group. ^#^ *P*<.05, ^##^ *P*<.01, ^###^ *P*<.001 as compared with pro100 group. GFAP, Glial fibrillary acidic protein; NeuN, Neuronal marker; IBA1, Ionized calcium‐binding adaptor molecule 1; TrkB, Tyrosine Kinase receptor B; PI3K, Phosphatidylinositol 3‐kinase; AKT, Protein kinase B; CREB, cAMP‐response element‐binding protein

Double immunofluorescence staining was performed to observe the co‐localization of TrkB on astrocytes (GFAP), neurons (NeuN), and microglia cells (IBA1) after edaravone intervened and these results showed that obviously expressed on astrocytes, neurons and microglia cells in eda3 + pro100 group (Figure 5A). Furthermore, in western blotting analysis, we found propofol‐induced apoptosis by up‐regulating the expression of cleaved caspase 3 at protein (Figure 5B, C, F3,8 = 14.59, *P<*0.001). Pre‐treatment with edaravone reduced apoptosis by shifting in the expression of cleaved caspase 3 (P<0.05). Taken together, these results supported our hypothesis that edaravone alleviated neural injury through phosphorylating TrkB.

We found when the TrkB was reduced phosphorylation by propofol induced (Figure 5B, C, F3.8 = 9.982, *P*<0.05), the downstream of PI3K (F_3,8_ = 18.93, *P*<0.001), AKT (F_3,8_ = 20.74, *P*<0.001) and CREB (F_3.8_ = 87.47, *P*<0.001) could also be inhibited compared to control group by propofol induced. We suggested that neural injury induced by propofol in hippocampal rats is closely related to TrkB/PI3K/CREB pathway.^7^, ^28^ With edaravone invention, eda3 + pro100 group animals showed significantly increased expression of phosphorylated TrkB (*P*<0.05), p‐PI3K (*P*<0.05), p‐AKT (*P*<0.001) and p‐CREB (*P*<0.001) compared with pro100 group. From these findings, we hypothesized that edaravone protects propofol‐induced neural injury and neuroinflammatory in developing rats by regulating TrkB/PI3K/CREB pathway.

### 3.5 7,8 DHF, a BDNF‐mimicking small compound and exogenous BDNF improve downstream TrkB pathway expression as same as edaravone in a rat model of excess propofol

To determine whether edaravone could influence BDNF, we further investigated the effect of the BDNF‐mimicking small compound 7,8 DHF and exogenous BDNF in propofol‐induced neural injury model. Activation of TrkB receptors was triggered by their autophosphorylation, which then activated downstream signalling cascades, also by phosphorylation.^19^ Both edaravone and 7,8 DHF treatments caused significantly be decreased expression of cleaved caspase 3 (Figure 5D, E, F 3, 8 = 12.13, *P*<0.05). Similarly, we demonstrated the significant rise in expression of phosphorylated TrkB (Figure 5D, E, F3,8 = 15.00, *P*<0.05), phosphorylated‐PI3K (F_3,8_ = 27.18, *P*<0.05; *P*<0.01), phosphorylated ‐AKT (F _3, 8_ = 25.56, *P*<0.05; *P*<0.01) and phosphorylated ‐CREB (F _3, 8_ = 60.41; *P*<0.01; *P*<0.001) in eda3 + pro100 group rats and 7,8 DHF + pro100 group rats when compared to pro100 group animals.

**FIGURE 6:**
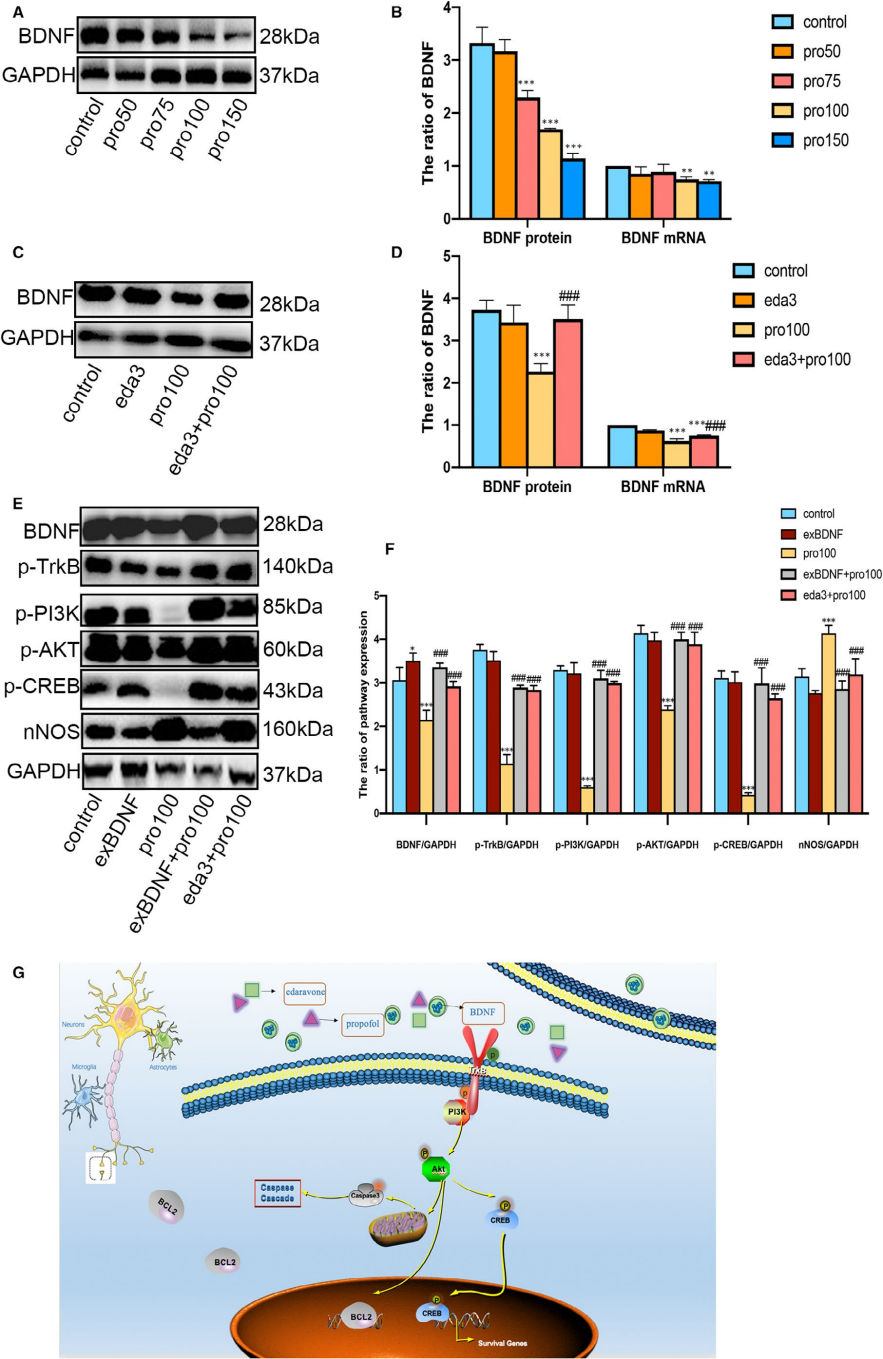
The role of BDNF in the entire conduction pathway A‐D, Western blot and RT‐qPCR detected endogenous BDNF expression in hippocampus of developing rats. E and F, Western blot detected exogenous BDNF expression in hippocampus of developing rats. G, signal pathway of edaravone neuroprotection against excess propofol. All data expressed as the mean ± SD. **P<.01, ***P<.001 as compared with control group. ^###^ *P*<.001 as compared with pro100 group. BDNF, Brain‐derived neurotrophic factor; TrkB, Tyrosine kinase receptor B; PI3K, Phosphatidylinositol 3‐kinase; AKT, Protein kinase B; CREB, cAMP‐response element‐binding protein

We found the mRNA and protein levels of BDNF in propofol concentration dependence induced rats were significantly decreased in comparison with control group (Figure 6A, B, F1, 16 = 89.43, *P*<0.001*; P*<0.01). As shown in Figure 6C, D, compared with pro100 group, the expression of BDNF mRNA and protein in eda3 + pro100 group was significantly elevated (*P*<0.001). Then, we explored whether edaravone and BDNF have similar effects, stimulating downstream pathways to exert neuroprotective effects. We designed the stereotaxic apparatus for exogenous injection of BDNF to interfere with propofol‐induced neural injury. We found that exogenous BDNF did increase the expression of the TrkB pathway similarly to edaravone. Interestingly, compared with the expression of nNOS in the propofol injury group, edaravone treatment and exogenous BDNF were significantly reduced, but it did not affect Long‐term potentiation (LTP) (Figure 6E, F). Taken together, these results supported our hypothesis that edaravone attenuated propofol‐induced neural injury in hippocampus of P7 rats by BDNF/TrkB/PI3K pathway.

## 4 DISCUSSION

The P7 SD rats are equivalent to 36‐week‐old newborn baby, which marks the peak of brain development.^26^, ^29^ Apparently, hippocampus is suitable as our research object because it plays a significant role in the pathophysiology of learning, memory, sentiment and nervous system diseases. Firstly, animal experiments revealed that propofol exposure could lead to neural injury, apoptosis and the increase of pro‐inflammatory factors IL‐6 and TNF‐α in rat hippocampal tissues. Cleaved caspase 3 is well‐recognized apoptosis‐related factors, the high expression of which is related to apoptosis cells.^2^, ^7^ The changes in Cleaved caspase 9 and BCL2 could cause mitochondrial injury. As a result, the expression of cleaved caspase 3 and cleaved caspase 9 was increased while that of BCL2 mRNA was reduced after propofol exposure, as discovered in our study. However, these negative effects can be reversed by the pre‐treatment of edaravone. Plenty of evidence has suggested that edaravone can exert neuroprotective effects by reducing the oxidative damage suffered by nerve cells and vascular endothelial cells.^10^, ^11^ In our study, it was observed that the most severe neural injury in hippocampus was manifested after propofol injection at 100mg/kg, but edaravone could also improve propofol‐induced neural apoptosis and neuroinflammatory. Besides, the molecular mechanism behind neuroprotective effects of edaravone remains clear.

Therefore, a study was conducted on the exact mechanism of its neuroprotective effects. BDNF is generated as the precursor, proBDNF, where it is cleaved to the developed mature BDNF. It is assumed that proBDNF and mature BDNF produce opposite physiological responses.^30^ The mature BDNF binds to TrkB so as to activate the downstream signalling pathways responsible for regulating gene expression encoding proteins involved in neural cell survival, axon and dendrite growth, as well as synapsis plasticity.^23^ In addition, the activation of PI3K/AKT/CREB signalling pathway is positively associated with the neuroprotective function that can cause neonatal hypoxia ischaemia.^7^, ^19^, ^31^ Furthermore, CREB is a protein that controls gene transcription and affects the regulation of downstream caspase 3 gene transcription and protein translation. Besides, its active form is a key factor in memory formation and the normal development of neurons (Figure 6G).^7^, ^15^, ^29^ Additionally, some results have indicated that edaravone can produce an excellent neuroprotective effect against the propofol‐induced neuron damage caused to the hippocampus, as evidenced by the increase of neural density and the reduced numbers of apoptosis cells, astrocytes and microglia cells in the hippocampal. When compared with the propofol concentration dependently reduced BDNF expression, the expression of BDNF at mRNA and protein level was found to increase in edaravone intervention group rats. In order to determine whether BDNF is involved in the protective mechanism of edaravone against propofol neural injury, exogenous BDNF (exBDNF) +pro100 group and BDNF‐mimicking small compound (7,8 DHF) +pro100 group were designed, respectively. Meanwhile, it was verified that similar to exBDNF + pro100 group and 7,8 DHF + pro100 group, edaravone activate phosphorylated TrkB and downstream pathway can produce a neuroprotective effect. Notably, it was also found out that the co‐localization of TrkB on astrocytes, neurons and microglia cells occurred after edaravone intervention, suggesting the expression of edaravone‐activated TrkB in astrocytes, neurons and microglia cells.^20^ Based on these findings, it is inferred that edaravone could alleviate the propofol‐induced neural injury in the hippocampus of developing rats through BDNF/TrkB/PI3K pathway.

Neuroinflammation can cause damage to neurons and lead to irreversible neural apoptosis, thus impairing brain functions.^32^ The evidence has indicated that the neuroinflammation in the hippocampus following propofol exposure might be a significant contributor to neural injury because IL‐6 and TNF‐α negatively regulate synaptic plasticity in the hippocampus.^33^ In our study, it was observed that edaravone produced a significant neuroprotective effect, for example, the suppressed up‐regulation of brain tissue expression of IL‐6 and TNF‐α, the attenuation of glial cells and the increase of neurons in the brain caused by propofol. Nitric oxide (NO) is an important molecule that is able to mediate neurotoxicity, neurotransmission and vasodilation. Under neuroinflammatory conditions, however, the excessive production of NO by nNOS can result in neuronal cell death and produce reactive nitrogen.^34^ While nNOS and NO signalling is associated with learning, memory and underlying long‐lasting synaptic plasticity.^35^ Learning and memory can be mediated by the neural plasticity.^26^ Among various synaptic plasticity mechanisms, long‐term enhancement (LTP) is considered a cell‐related factor in learning and memory.^36^, ^37^ In our study, it was demonstrated that both edaravone and exogenous BDNF could reverse the increase of nNOS caused by excessive propofol. However, we failed to find out that the propofol‐induced neural injury model could have an impact on long‐term learning and memory,^38^ which is potentially attributed to the fact that we designed concentration of propofol and a single injection of propofol caused short‐term neurotoxicity.

In conclusion, edaravone can produce neuroprotective effects against propofol‐induced neural injury in the hippocampus of developing rats through BDNF/TrkB/PI3K pathway.

## CONFLICTS OF INTEREST

The authors have declared no conflict of interest.

## AUTHOR CONTRIBUTION

**Yangliang Yang:** Conceptualization (equal); Data curation (equal); Formal analysis (equal); Funding acquisition (equal); Investigation (equal); Methodology (equal); Project administration (equal); Resources (equal); Software (equal); Supervision (equal); Validation (equal); Visualization (equal); Writing‐original draft (equal); Writing‐review & editing (equal). **Jing Yi:** Conceptualization (equal); Data curation (equal). **Mengzhi Pan:** Conceptualization (equal); Data curation (equal). **Baoji Hu:** Conceptualization (equal); Data curation (equal). **Hongwei Duan:** Conceptualization (equal); Data curation (equal); Formal analysis (equal); Funding acquisition (equal); Investigation (equal); Methodology (equal); Project administration (equal); Resources (equal).

## Supporting information

Table S1

## Data Availability

Data supporting the findings of our study are obtained from corresponding authors upon reasonable request.
