# Supplementary material for: Edaravone alleviated propofol‐induced neural injury in developing rats by BDNF/TrkB pathway
Source: J Cell Mol Med. 2021 May 1;25(11):4974–87. doi: 10.1111/jcmm.16422 (PMC8178254; doi:10.1111/jcmm.16422)
Supplement: Supplementary file 1 — Table S1 [file JCMM-25-4974-s001.docx]

|  |  | PH | SaO_2_ | PaO_2_ | PaCO_2_ |
| --- | --- | --- | --- | --- | --- |
| **Part 1** | Control | 7.34± 0.01 | 96.90±1.37 | 100.25±2.94 | 40.35±2.17 |
|  | Pro50 | 7.33±0.02 | 95.64±1.22 | 99.04±5.67 | 41.19±2.53 |
|  | Pro75 | 7.35±0.01 | 96.84±1.02 | 97.88±4.34 | 37.94±2.38 |
|  | Pro100 | 7.35±0.01 | 95.15±0.19 | 100.84±8.55 | 39.76±3.84 |
| **Part 2**  **Part 3**  **Part 4** | Pro150  Control  Pro100  Eda1+pro100  Eda3+pro100  Eda5+pro100  Control  Eda3  Pro100  Eda3+pro100  Control  Pro100  7,8DHF+pro100 | 7.34±0.02  7.35±0.02  7.36±0.01  7.35±0.01  7.36±0.01  7.34±0.02  7.36±0.03  7.36±0.03  7.38±0.02  7.35±0.03  7.34±0.04  7.36±0.02  7.35±0.02 | 96.28±1.42  95.38±2.42  96.45±3.02  94.89±3.46  95.14±3.15  96.29±2.07  94.78±3.41  95.78±3.43  93.47±3.88  93.93±2.96  97.39±1.34  95.98±1.79  95.38±2.43 | 99.64±7.09  102.63±7.37  99.39±5.41  101.45±7.30  98.30±8.39  103.49±7.39  101.52±4.59  102.67±4.87  99.63±5.58  96.84±2.72  100.37±4.29  102.39±3.01  103.98±1.20 | 39.09±1.99  40.29±2.73  38.28±4.20  39.71±2.84  39.98±3.07  41.24±2.93  39.30±2.28  40.56±2.24  39.48±1.76  39.80±2.63  37.89±3.24  40.59±1.30  38.29±2.04 |
| **Part 5** | Eda3+pro100  Control  Pro100  ExBDNF+pro100  Eda3+pro100 | 7.34±0.03  7.35±0.04  7.40±0.03  7.36±0.03  7.42±0.02 | 97.32±0.92  96.90±1.25  95.86±4.30  96.06±4.31  98.11±2.70 | 99.25±6.44  104±5.32  107.62±3.32  105.36±5.46  108.93±1.28 | 38.91±2.53  40.72±4.04  36.29±3.02  37.09±2.47  41.63±3.79 |

Supplementary table 1 Arterial blood gas analysis

It was given values as mean ± SD each group. PH = arterial hydrogen ion concentration; PaCO2 = arterial carbon dioxide tension; PaO2 = arterial oxygen partial pressure; SaO2 = arterial oxygen saturation. *P < .05 vs control group by one ANOVA followed by Dunnet’s post hoc test.
